# Ubiquitin specific peptidase 11 knockdown slows Huntington’s disease progression via regulating mitochondrial dysfunction and neuronal damage depending on PTEN-mediated AKT pathway

**DOI:** 10.1186/s10020-024-01061-w

**Published:** 2025-01-08

**Authors:** Bai Gao, Yuchen Jing, Xi Li, Shuyan Cong

**Affiliations:** 1 Department of Neurology, Shengjing Hospital of China Medical University, Shenyang, China; 2 Department of Vascular Surgery, The First Hospital of China Medical University, Shenyang, China

**Keywords:** Huntington’s disease (HD), Ubiquitin specific peptidase 11 (USP11), Phosphatase and tensin homolog (PTEN), Neuronal apoptosis, Mitochondria

## Abstract

**Background:**

Mitochondrial dysfunction and neuronal damage are major sign of cytopathology in Huntington’s disease (HD), a neurodegenerative disease. Ubiquitin specific peptidase 11 (USP11) is a deubiquitinating enzyme involved in various physiological processes through regulating protein degradation. However, its specific role in HD is unclear.

**Methods:**

To interfere with USP11 expression, adeno-associated viruses 2 containing USP11-specific shRNA were injected into the bilateral striatum of 12-week-old R6/1 and WT mice. In vitro, the inducible PC12 cell model of HD was used in which the expression of an N-terminal truncation of huntingtin, with either wild type (Q23) or expanded polyglutamine (Q74) can be induced by the doxycycline. USP11 was knocked down to study its role in HD. The protein expression patterns in Q74 cells were quantified by label-free proteomics to further explore the target protein of USP11. Detecting the association between USP11 and Phosphatase and Tensin Homolog (PTEN) through Co-IP.

**Results:**

Herein, USP11 was found to be upregulated in the striatum of R6/1 mice (an HD model with gradual development of symptoms) in an age-dependent manner. The spontaneous HD was alleviated by silencing USP11, as evidenced by improved locomotor activity and spatial memory, attenuated striatal atrophy in R6/1 mice, reduced accumulation of mutant huntingtin protein, and restored mitochondrial function in vitro and in vivo. The results of label-free proteomics revealed a significant change in the protein expression profile. Through functional enrichment, we focused on PTEN, known as a negative regulator of the AKT pathway. We demonstrated that USP11 downregulation promoted ubiquitination modification of PTEN and activated the AKT pathway, and PTEN overexpression reversed the effects of USP11 knockdown.

**Conclusions:**

Collectively, USP11 knockdown protects R6/1 mouse neurons from oxidative stress by alleviating mitochondrial dysfunction, thereby preventing the HD progression. This is achieved by inhibiting PTEN expression, which in turn activates the AKT pathway. This study suggests that USP11-PTEN-AKT signaling pathway may be a new attractive therapeutic target for HD.

## Introduction

Huntington’s disease (HD) is a prevalent autosomal dominant neurodegenerative disorder caused by an expansion of a cytosine-adenine-guanine (CAG) sequence in the HD gene coding region (MacDonald et al. 1993). Normal individuals have approximately 20 CAG repeat in the HD gene. As the number of CAG repetitions increases, the possibility of polyglutamine expansion in the N-terminal region of the protein also increases. This results in the misfolding of huntingtin (HTT) and the formation of insoluble oligomers. If this repetition occurs more than 36 times, it is considered a pathogenic mutation. The repetitive sequence of CAG encodes the mutant huntingtin (mHtt) (Semaka et al. 2013). Current treatment of HD focuses largely on managing symptoms. However, as of now, the Food and Drug Administration (FDA) has not approved any disease-modifying treatments for HD (Pan and Feigin 2021). Therefore, revealing the molecular mechanisms of HD and identifying potential therapeutic targets are promising strategies for treating patients with HD.

Ubiquitin-specific processing proteases were reported to be involved in the regulation of neuroinflammation (Jiang et al. 2017; Zhu et al. 2015). Ubiquitin specific peptidase (USP) 11 is a deubiquitinating enzyme that belongs to the ubiquitin-specific processing protease family (Jacko et al. 2016; Rong et al. 2022; Zhang et al. 2021). Amongst the numerous USP, the USP11 has a preferential high expression level in the brain compared to other USP proteins based on the Human Protein Atlas database analysis. Allen Brain Atlas showed that USP11 mRNA was prevalently expressed in C57BL/6J mouse brains. The upregulation of USP11 exacerbated traumatic brain injury in rats by inducing neurological impairment and neuronal apoptosis (Fang et al. 2023). After suppressing the expression of USP11, the neural damage in rats with cerebral hemorrhage was alleviated, accompanied by a reduction in neuronal apoptosis, microglial polarization, and inflammation (Zhang et al. 2021; Xu et al. 2016). This evidence suggests a potential connection between USP11 and neurological disease, but our understanding of the relationship between USP11 and HD is limited.

Post-translational modifications enhanced protein versatility by increasing phosphorylation, ubiquitination, methylation, and other processes (Basak et al. 2016). As a deubiquitinating enzyme, USP11 has been reported to be involved in regulating neural function by mediating the deubiquitination modification of downstream proteins to increase their stability, such as Beclin 1 (Rong et al. 2022). Based on this, this study analyzed the protein expression patterns in Q74 cells using label-free proteomics. PTEN, a phosphatase that removes phosphate groups from its bound substrates, was screened out based on the results of functional enrichment, and its interaction with USP11 was further explored in HD progression. AKT Serine/Threonine Kinase (AKT) is a crucial protein regulated by PTEN, an important tumor suppressor that promoted apoptosis by dephosphorylating AKT (Piguet and Dufour 2011; Deng et al. 2020). Knockdown of PTEN protected hippocampal neurons from oxidative stress damage (Zhu et al. 2006). Promoting AKT phosphorylation inhibited striatal neuronal damage caused by misfolded HTT (Humbert et al. 2002).

This study reported that the R6/1 mouse and WT mouse from F1 progeny were obtained by crossbreeding male R6/1 mice with female C57BL/6J mice. The regulatory effect of USP11 on HD was investigated by knocking down USP11 in the striatum of WT and HD mice. The effect of USP11 on protein expression was evaluated using label-free proteomics in doxycycline (Dox)-induced PC12 cells employing the Tet-on system in vitro. Through the enrichment of genes associated with phosphorylation and cell growth, the focus was placed on PTEN, which was recognized as a negative regulator of the AKT pathway. Furthermore, the regulatory mechanism of USP11 and PTEN on mitochondrial dysfunction and neuronal damage was investigated in vitro. Our study revealed the mechanism through which USP11 and PTEN safeguard neurons in HD.

## Materials and methods

### Antibodies

The primary antibodies used for immunofluorescence in myocardial tissues included mHtt antibody (Sigma-Aldrich, St. Louis, MO, USA), USP11 antibody (Santa Cruz, Dallas, TX, USA), Neuronal Nuclei (NeuN) antibody (Abcam, Boston, MA, USA), PTEN antibody (Proteintech, Rosemont, IL, USA), p-AKT antibody (Affinity, Cincinnati, OH, USA), and AKT antibody (Affinity). FITC-conjugated secondary antibody (goat anti-rabbit IgG) and Cy3-conjugated secondary antibody (goat anti-mouse IgG) were from Proteintech. For the Western blot analysis, the primary antibodies used included the USP11 antibody purchased from Santa Cruz, the mHtt antibody purchased from Sigma-Aldrich, the Bax, Bcl-2, and Ubi antibody obtained from Wanleibo (Shenyang, China), the cytochrome c antibody, the PTEN antibody, the COX IV antibody, and the β-actin antibody obtained from Proteintech. The HRP-conjugated secondary antibodies used were goat anti-rabbit IgG and goat anti-mouse IgG (Proteintech).

### Adeno-associated virus (AAV)

To downregulate USP11 expression, oligonucleotides targeting the mouse USP11 (shUSP11: 5′-GGTGGAAGTGTACCCACTAGA-3′) or the sequence without any predicted target gene were cloned into the pAAV2-CMV-U6 Track vector. Then, the pAAV2-CMV-U6 Track vector was co-transfected with the pHelper plasmid and pRC2-mi342 plasmid into AAV-293 cells (iCell Bioscience Inc, Shanghai, China) using Lipofectamine 3000 (Invitrogen, Carlsbad, CA, USA) to generate infectious AAV viral particles containing a plasmid with USP11 shRNA and NC shRNA.

### Animal experiments

Male R6/1 mice (8-week-old male, C57BL/6J background) purchased from the Jackson Laboratory (RRID: IMSR_JAX:006471; Main Harbor, NY, USA), expressing exon1 of the human HTT gene, were crossbred with female C57BL/6J mice. Genotypes of the F1 individuals were determined using PCR analysis.

### Part 1

A total of 36 littermates of F1, including 18 R6/1 mice and 18 WT mice, were used in this part. The WT mice and R6/1 mice were kept for 8, 16, and 20 weeks, respectively. Subsequently, the mice were euthanized, and the striatum was collected.

### Part 2

Thirty-six WT mice and 36 R6/1 mice were divided into two groups (shNC and shUSP11, n = 18), respectively. Three mice were required to complete all the analyses. The mice were injected with 2 µl of AAV-2 containing USP11-specific shRNA (shUSP11) and NC shRNA (shNC) at a concentration of 1.53 × 10^9^ genomic copies. The injections were performed in the striatum using coordinates relative to bregma as previously described (Creus-Muncunill et al. 2018): (1) anteroposterior (AP), + 0.8; mediolateral (ML), + 1.8; and dorsoventral (DV), 2.9 mm, and (2) AP, + 0.3; ML, + 2; and DV, 3 mm below the dural surface, with the incisor bar positioned 3 mm above the interaural line. After 4 weeks of injections, the motor ability and working memory of each group were evaluated using the open field test and Y-maze test, as previously reported with slight modification (Angeles-López et al. 2021; Ruskin et al. 2011). The mice were allowed to freely explore the two open arms for 5 min. After an interval of 10 min, the test was started. In the testing phase, the mice were allowed to explore all three arms for 5 min. The time spent in the novel arm divided by the time spent in all three arms × 100 was calculated to represent a preference index. Subsequently, the mice were euthanized, and the striatum was collected. All animal experiments have been approved by the Medical Ethics Committee of Shengjing Hospital of China Medical University and followed the Guide for the Care and Use of Laboratory Animals (Eighth Edition).

### Cell culture

Inducible rat PC12 cells expressing an exon 1 fragment of HTT with either 23 (Q23) or 74 (Q74) glutamine repeats, fused to Green Fluorescent Protein (GFP), were maintained in DMEM culture medium (Servicebio, Wuhan, China) supplemented with 10% fetal calf serum (Tianhang Biotech, Huzhou, China) and cultured at 37 ℃ with 10% CO_2_. To induce the expression of HTT, the cells were treated with Dox (1 µg/ml) (Macklin, Shanghai, China) for durations of 0, 1, 3, and 6 days. The induced cells were exposed to cycloheximide (CHX) (Aladdin, Shanghai, China) for 0, 0.5, 1, and 2 h, respectively, to inhibit protein synthesis.

### Cell transfection

The sequence of shUSP11 or shNC was cloned into a plasmid vector. The cells were transfected with the plasmids using Lipofectamine 3000. The cell viability was detected using a CCK-8 cell proliferation detection kit (KeyGEN, Nanjing, China).

### Real-time qPCR

The samples were homogenized in TRIpure lysis buffer (BioTeke Bio., Beijing, China) to extract total RNA. The RNA concentration was analyzed using a UV spectrophotometer (NANO 2000, ThermoFisher Scientific, Pittsburgh, PA, USA). The cDNA synthesis was performed using BeyoRT II M-MLV reverse transcriptase (BeyotimeBiotech, Shanghai, China). The mRNA expression levels were evaluated using real-time qPCR with a fluorescent quantitative PCR instrument (Exicycler 96, Bioneer, Daejeon, Korea). The results were calculated using the 2^–△△CT^ method. The primer sequences are as follows: Rat USP11, 5′-GGCAGCCTATGTCTTGT-3′ (F), 5′-GATGTCAGAGTTGGGTGTA-3′ (R). Mus USP11: 5′-GATGTACCGACTTTCACG-3′ (F), 5′-GCTGTTGTCTAAGAGGGAT-3′ (R). Rat β-actin, 5′-GGAGATTACTGCCCTGGCTCCTAGC-3′ (F), 5′-GGCCGGACTCATCGTACTCCTGCTT-3′ (R). Mus β-actin, 5′- CATCCGTAAAGACCTCTATGCC-3′ (F), 5′-ATGGAGCCACCGATCCACA-3′.

### Protein extraction and western blot

For the total protein analysis, the sample was lysed for 30 min on ice in RIPA lysis buffer (Proteintech) containing 1% protease inhibitor (Proteintech). After centrifugation, the lysate was separated, and the supernatant was collected. The mitochondrial protein was extracted using a mitochondrial isolation and protein extraction kit (Proteintech), following the provided instructions. A BCA protein concentration determination kit (Proteintech) was used to measure the protein concentration. The protein sample was separated using SDS-PAGE and then transferred from the gels onto polyvinylidene difluoride (PVDF) membranes (Thermo Fisher Scientific, Pittsburgh, PA, USA). The membranes were then blocked in 5% skim milk (Proteintech). The blocked membranes were incubated with the primary antibodies (USP11 antibody, 1:300; cytochrome c antibody, 1:2000; mHtt antibody, 1:500; PTEN antibody, 1:5000; Ubi antibody, 1:1000; COX IV, 1:5000; β-actin, 1:20000) overnight at 4 °C. Incubation with antibodies against β-actin or COX IV was performed to obtain loading controls. After the primary antibody incubation, the membranes were washed and then incubated for 40 min at 37 °C with the secondary antibodies (1:10000). Finally, the blot was visualized using the hypersensitive ECL chemiluminescence test kit (Proteintech).

### Co-immunoprecipitation (Co-IP)

The cells were lysed in a lysis solution for 30 min. AminoLink^®^ coupling resin cross-linked with the antibody was washed using lysis buffer, and the liquid flowed through the resin was discarded. Next, the lysate was added into the resin and incubated overnight 4 ℃. Subsequently, then the resin was washed with lysis buffer. Elution buffer was added into the resin to release the precipitate from binding to the resin. The eluate was then was using the western blot procedure.

### Immunofluorescence

The tissues were embedded in paraffin and then cut into 5-µm sections, which were dewaxed and boiled in an antigen retrieval solution for 10 min. The cells grown on coverslips were fixed with 4% paraformaldehyde and permeabilized using 0.1% Triton X-100 (BeyotimeBiotech). The slices were blocked in 1% BSA (Sangon, Shanghai, China) for 15 min. The blocked slices were then incubated with the primary antibodies (1:50) overnight at 4 °C and with secondary antibodies (1:200) for 1 h at 37 °C. Finally, the nuclei were counterstained using DAPI (Aladdin Reagents Co. Ltd., Shanghai, China).

### Terminal deoxynucleotidyl transferase dUTP nick end labeling (TUNEL) assay

The cells grown on coverslips and the tissue sections were permeabilized using 0.1% Triton X-100. The TUNEL assay was conducted using the In Situ Cell Death Detection Kit (Roche, Basel, Switzerland), according to the manufacturer’s instructions. The nuclei were counterstained with DAPI. For the TUNEL assay combined with immunofluorescence for NeuN, the TUNEL assay was performed first, and the slides were then rinsed before performing immunofluorescence for NeuN using the previously described methods.

### Caspase-3 activity assay

The protein was extracted using the lysis buffer, and its concentration was determined using the Bradford protein concentration assay kit (BeyotimeBiotech). The Caspase-3 activity was detected using the Caspase-3 activity detection kit (BeyotimeBiotech) according to the manufacturer’s instructions. Optical density was measured using a microplate reader (ELX-800, BioTek, Winooski, VT, USA).

### Hematoxylin eosin (H&E) staining

The dewaxed sections were stained with hematoxylin (Solarbio, Beijing, China) for 5 min and then cleared in distilled water. Afterward, the sections were washed and counterstained with Eosin (Sangon) for 3 min, then washed and photographed for later analysis of the lateral ventricle area.

### Flow cytometry (FCM)

The cells were mixed with Annexin V-Light 650 and PI dye (Wanleibo) and incubated in the dark for 15 min. Apoptosis was examined using a flow cytometer (NovoCyte, Agilent, Santa Clara, CA, USA).

### Reactive oxygen species (ROS) detection

MitoSOX staining was performed to assess the level of ROS in cells. The cells were incubated with 5-µM MitoSOX dye at 37 ℃ for 10 min and then observed under a fluorescence microscope.

### Mitochondrial membrane potential

Mitochondrial membrane potential was detected using a Mito-Tracker Red CMXRos red fluorescent probe. In detail, 100 nM Mito-Tracker Red CMXRos staining solution (Maokang Bio, Shanghai, China) was added to the cells and incubated at 37 ℃ for 30 min. The excess dye was then discarded and DMEM medium was added to the cells. The cells were imaged under a fluorescence microscope.

### Transmission electron microscope (TEM)

The sample was immersed in acetone and an embedding agent (SPI, West Chester, PA, USA), and then polymerized at 60 °C for 48 h. Subsequently, the sample was cut into 60–80 nm sections. The slices were stained with a 2% uranium acetate solution in saturated alcohol for 8 min in the dark. After washing, the sections were stained with a 2.6% lead citrate solution in carbon dioxide for 8 min. The stained sections were observed using a TEM (H-7650, Hitachi, Tokyo, Japan).

### Label-free proteomics

Protein samples were incubated with dithiothreitol (DTT) for 1 h at 37 °C to break down disulfide bonds. Then, iodoacetamide was added into samples and incubated for 45 min at room temperature in the dark. Samples were diluted with NH_4_HCO_3_ and incubated overnight with trypsin at 37 ℃ for enzymatic digestion. Formic acid was used to stop the reaction. The sample was desalted using a C18 column. The peptide mixture was separated using the RIGOL L-3000 high-performance liquid chromatography system (Beijing RIGOL Technology Co., Ltd., Beijing, China) and analyzed using the ORBITRAP ECLIPSE mass spectrometer (Thermo Fisher Scientific). The gene ontology (GO) annotation and enrichment analysis were conducted on the differentially expressed proteins.

### Bioinformatic analysis

Molecular simulated docking of USP11 and PTEN was generated using the GRAMM website (Singh et al. 2024). The ubiquitination modification sites of PTEN were predicted using the MusiteDeep website (Wang et al. 2020).

### Data analysis

All data were presented as mean ± standard deviation (SD). Data analysis was performed using GraphPad Prism 9.5 (GraphPad Software Inc., La Jolla, CA, USA). The t-test or ANOVA was used to compare the differences in means. Differences were considered statistically significant at *p* < 0.05.

## Result

### USP11 was highly expressed in mice with HD

**Fig. 1:**
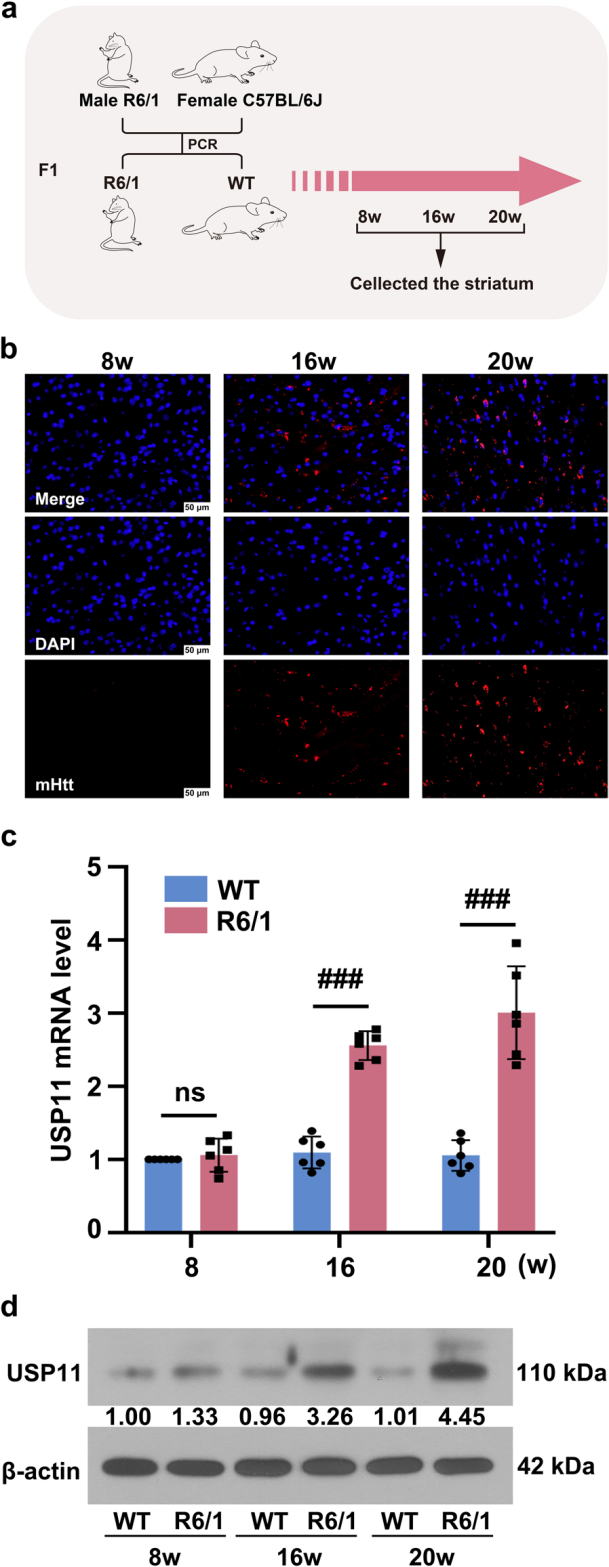
**a** The male R6/1 mice were crossbred with female C57BL/6J mice to obtain the F1 (WT, R6/1). **b** The expression of mHtt was detected using immunofluorescence staining. **c** The USP11 mRNA expression was analyzed using real-time qPCR. **d** The USP11 protein level was analyzed using western blotting. ns, p > 0.05, ^###^p < 0.001. mHtt: mutant huntingtin; USP11: Ubiquitin specific peptidase 11

R6/1 transgenic mice and the WT mice were obtained by crossing male R6/1 with female C57 mice, as indicated in Fig. 1a. The expression of mHtt in the striatum of R6/1 mice was detected using immunofluorescence. As shown in Fig. 1b, mHtt expression was not observed in the striatum of 8-week-old R6/1 mice. The mHtt aggregates were present in the striatum of 16-week-old R6/1 mice, and was further increased in 20-week-old R6/1 mice. The expression of USP11 in the striatum of R6/1 mice was higher than that of WT mice and increased with the age of the mice (Fig. 1c, d).

### Knocking down USP11 improved the locomotor abilities and working memory of mice with HD

**Fig. 2:**
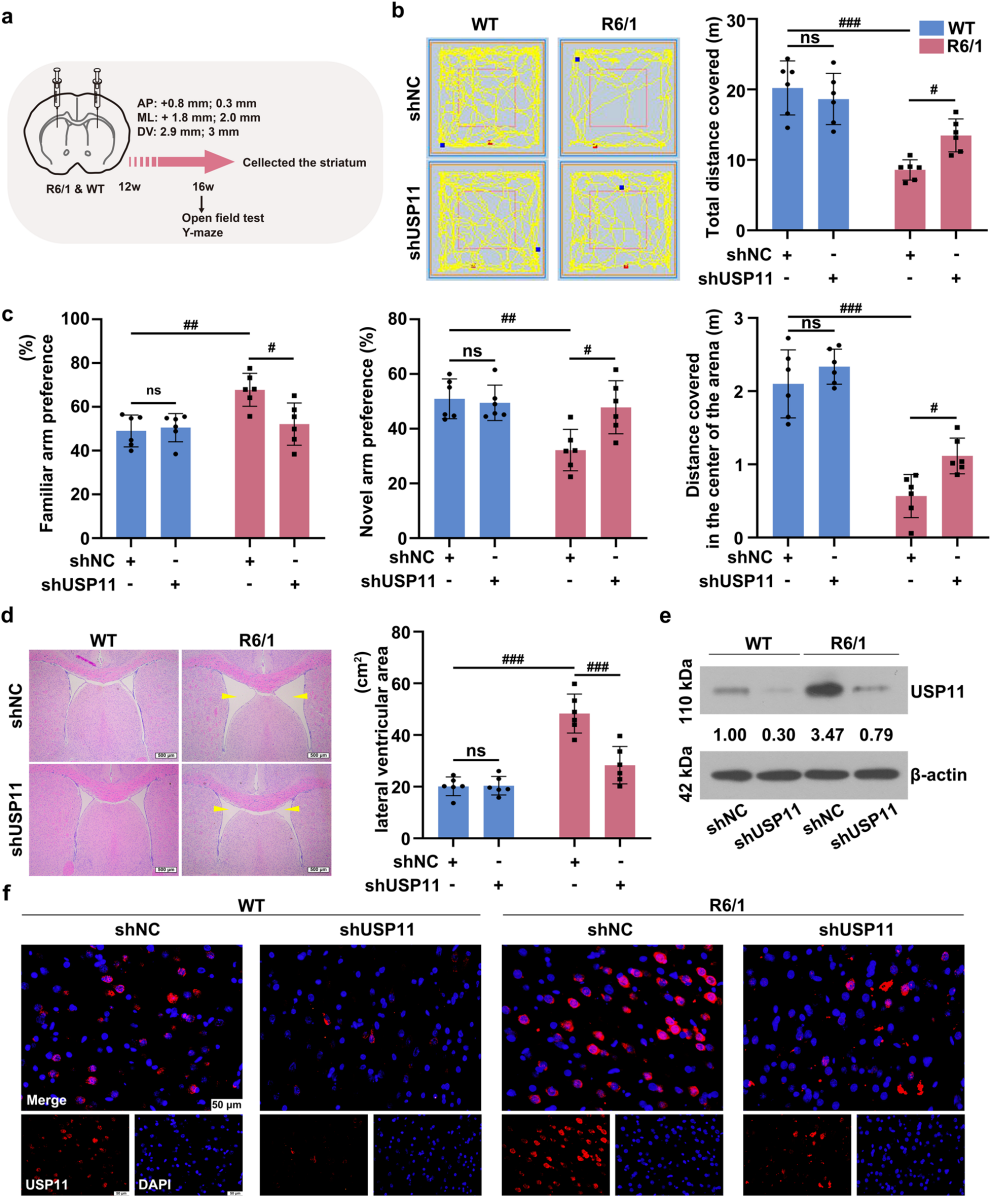
**a** The adeno-associated virus carrying USP11-specific shRNA was injected into the striatum. **b** The results of the open field test. **c** The results of the Y-maze test. **d** The results of H&E staining and quantitative measurement of the lateral ventricular area in the striatum tissue (the yellow arrows in the images represented the location of the lateral ventricle). **e** The USP11 protein level was analyzed using western blotting. **f **The expression of USP11 in the striatum tissue was detected using immunofluorescence staining. ns, p > 0.05, ^#^p < 0.05, ^##^p < 0.01, ^###^p < 0.001

Based on the observed changes in USP11 expression in the striatum, AAV-2 containing USP11-specific shRNA was injected into the striatum of WT and R6/1 mice to downregulate USP11 expression (Fig. 2a). After 16 weeks, the results of the open field test showed that the total distance covered by R6/1 mice was decreased (Fig. 2b). Interestingly, the distance in the center of the arena covered by R6/1 mice was shortened. USP11 knockdown increased the motion distance of R6/1 both the total distance and the distance covered in the center of the arena. However, USP11 silence did not have a significant effect on the WT mice. The results of the Y-maze test indicated that the residence time in the novel arm of R6/1 mice decreased, while it in the familiar arm increased (Fig. 2c). Silencing of USP11 promoted the exploration of new arms in R6/1 mice. H&E staining results (Fig. 2d) showed the lateral ventricular area enlargement and striatum atrophy in R6/1 mice. However, USP11 knockdown reduced the lateral ventricular area, as indicated by the yellow arrows, and alleviated the atrophy of striatum. The results of western blot and immunofluorescence staining showed that without targeted intervention of USP11 expression, the expression of USP11 in the striatal tissues of R6/1 mice is higher than that of WT mice (Fig. 2e and f). Reduced USP11 expression was observed in the striatal tissues of shUSP11 group in both WT and R6/1 mice, suggesting that the injection of USP11-targeted AAV2 successfully knocked down USP11 expression in the mouse striatum.

### Downregulation of USP11 reduced mHtt aggregate formation and neuronal death

**Fig. 3:**
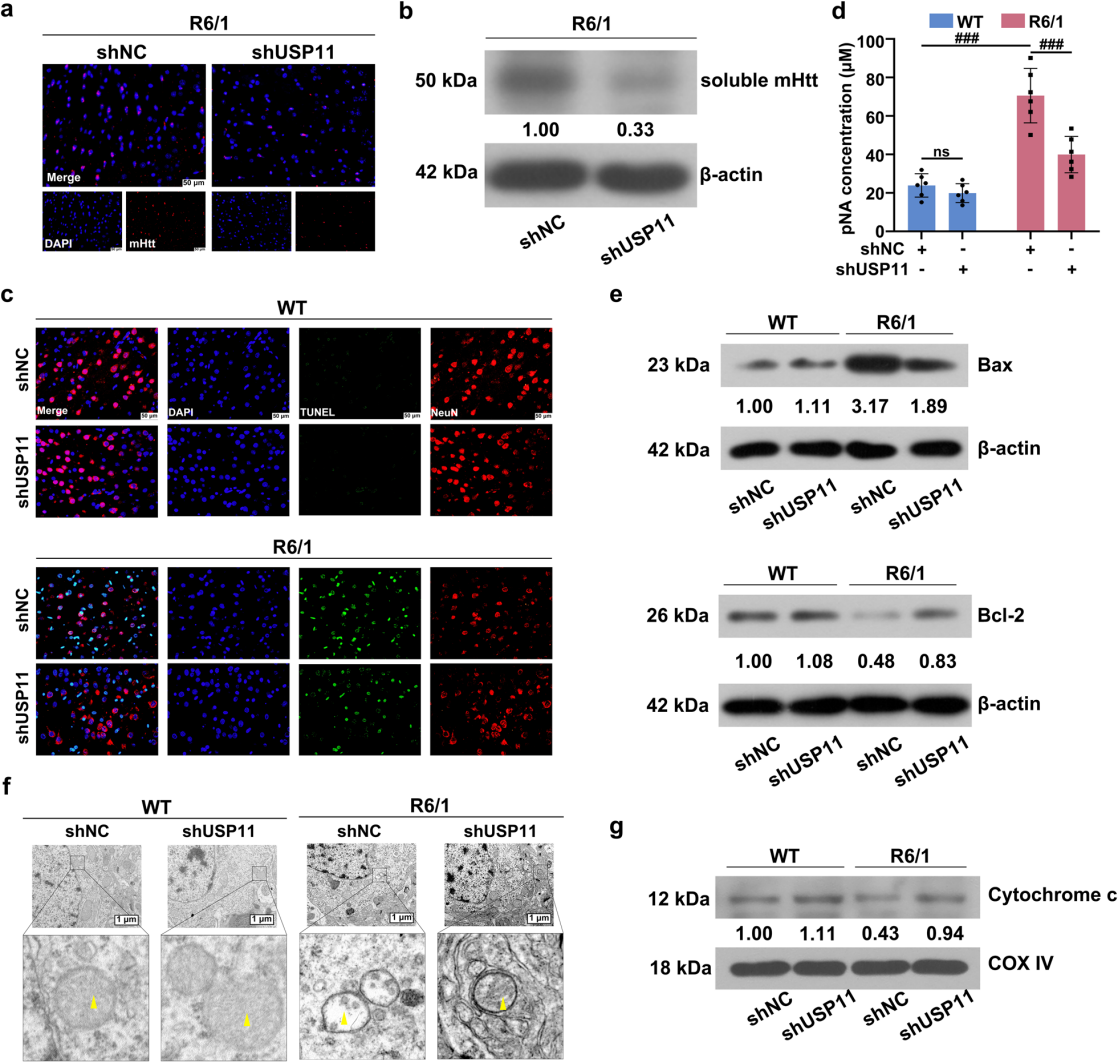
**a** The expression of mHtt aggregates was detected using immunofluorescence staining. **b **The soluble mHtt protein level was analyzed using western blotting. **c **NeuN immunofluorescence staining and TUNEL staining of neurons. **d** The caspase-3 activity was detected using the kit. **e** The protein level of Bax and Bcl-2 was analyzed using western blotting. **f **The mitochondria were observed using the transmission electron microscope (the yellow arrows in the images represented the mitochondrial cristae). **g** The cytochrome c protein level in mitochondria was analyzed using western blotting. ns, p > 0.05, ^###^p < 0.001. mHtt: mutant huntingtin; Bax: BCL2 Associated X Apoptosis Regulator; Bcl-2: BCL2 Apoptosis Regulator

As shown in Fig. 3a, a lot of mHtt aggregates was observed in the R6/1 mice, however, the USP11 knockdown cleared most of the mHtt aggregates. Western blot results showed that soluble mHtt was reduced by USP11 knockdown (Fig. 3b). As shown in Fig. 3c, the R6/1 groups showed a large number of TUNEL-positive neurons, which were reduced by USP11 silencing. For some markers, in the R6/1 mice, Caspase-3 activity and Bax expression were increased, and Bcl-2 expression was decreased. USP11 knockdown reversed the levels of these markers (Fig. 3d and e).

###  Downregulation of USP11 prevented mitochondrial damage

Mitochondrial ultrastructure in striatum of the WT mice is intact. However, the absence of mitochondrial cristae can be observed in the R6/1 mice. USP11 knockdown prevented the damage of mitochondria structure. The yellow arrows indicate the mitochondrial cristae. The expression of cytochrome c in mitochondria was reduced in the R6/1 groups (Fig. 3g). USP11 silence increased the level of cytochrome c in the striatum of R6/1 mice.

### Downregulation of USP11 reduced apoptosis in Q74 cells

**Fig. 4:**
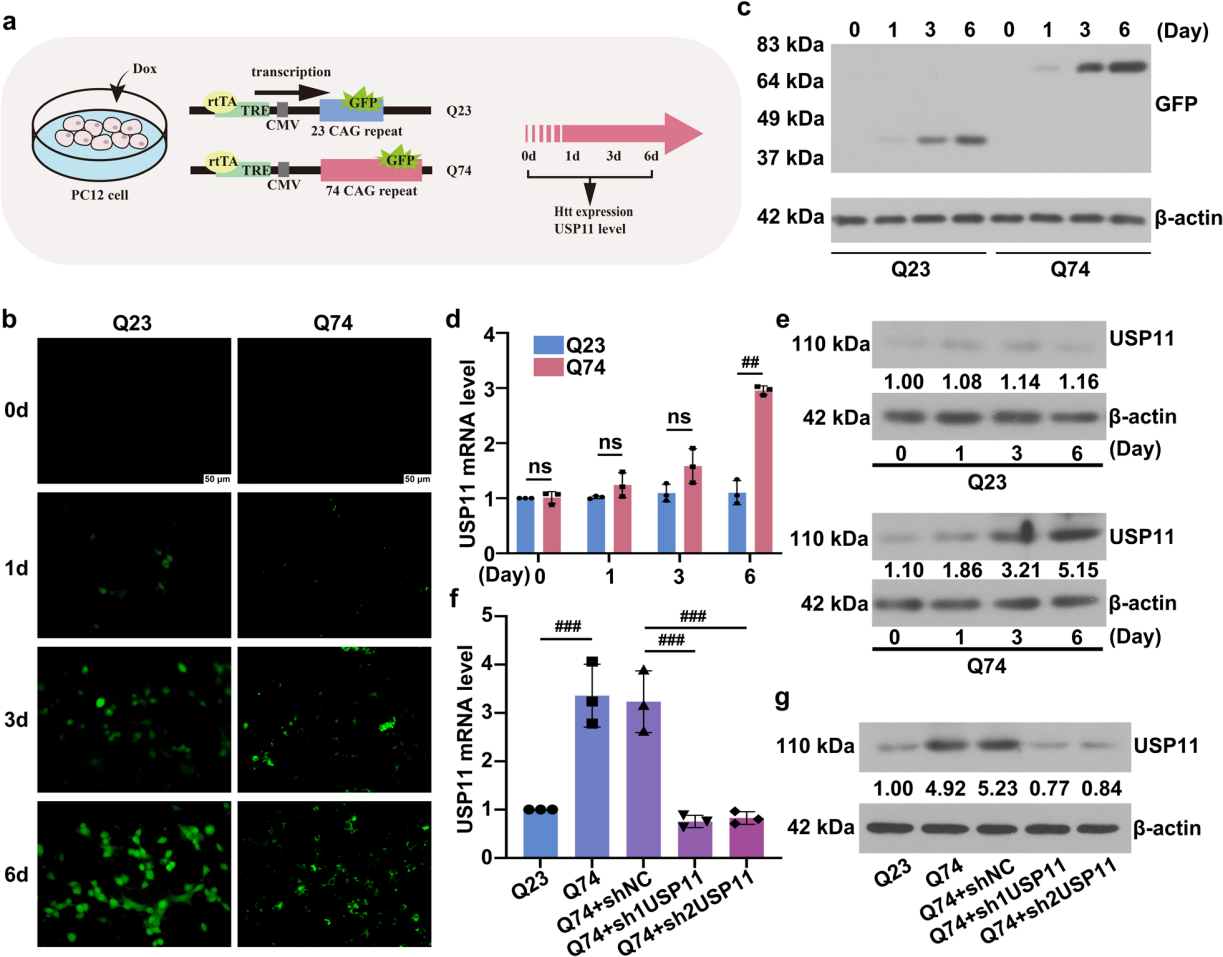
**a** The HD cell model was established by inducing PC-12 cells based on the Tet-on system. **b** The induced cells were observed using a fluorescence microscope. **c** The GFP expression was analyzed using western blotting. **d** The USP11 mRNA expression was analyzed using real-time qPCR. **e** The USP11 protein level was analyzed using western blotting. **f** The USP11 mRNA expression was analyzed using real-time qPCR after transfection. **g** The USP11 protein level was analyzed using western blotting after transfection. ns, p > 0.05, ^##^p < 0.01, ^###^p < 0.001. HD: Huntington’s Disease; USP11: Ubiquitin specific peptidase 11; GFP: Green Fluorescent Proteins

**Fig. 5:**
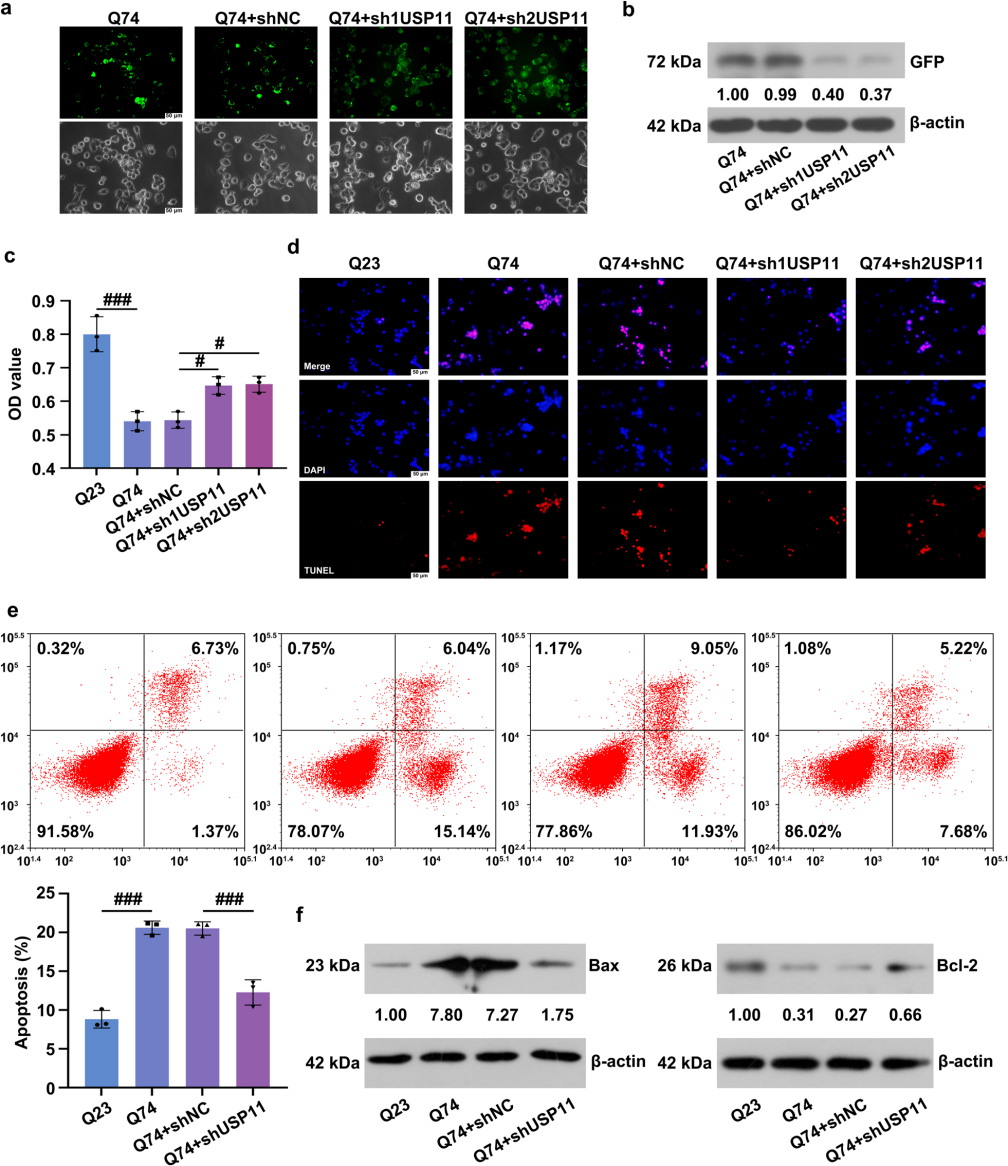
**a** The expression of mHtt aggregates was observed using a fluorescence microscope. **b** The GFP expression was analyzed using western blotting. **c** The cell viability was detected using the CCK-8 assay. The apoptosis was analyzed using a TUNEL assay (**d**) and flow cytometry (**e**). **f** The protein level of Bax and Bcl-2 was analyzed using western blotting. ns, p > 0.05, ^#^p < 0.05, ^###^p < 0.001. mHtt: mutant huntingtin; GFP: Green Fluorescent Proteins; Bax: BCL2 Associated X, Apoptosis Regulator; Bcl-2: BCL2 Apoptosis Regulator

To confirm these results in vitro, an inducible HD cell model was established. The GFP tag was fused to the N-terminal of HTT exon 1, which contained 23 CAG repeats and 74 CAG repeats in PC-12 cells, respectively (Fig. 4a). The GFP tag was expressed after induction by a culture in the presence of Dox, based on the Tet-on system. The HTT expression was determined by the fluorescence intensity. Fluorescence microscopy observations revealed that the HTT expression increased as the induction time extended, reaching its peak after 6 days of induction (Fig. 4b). In Q23 cells, HTT showed a diffuse staining, while in Q74 cells, large aggregates were visible. The expression of GFP was examined. It can be observed that the bands from Q74 cells exhibited a higher molecular weight, indicating the fusion expression of mHtt and GFP (Fig. 4c). In the Q74 cell, the expression of USP11 increased as the induction time was extended (Fig. 4d, e). Conversely, the expression of USP11 in the Q23 cells did not exhibit a significant change during the culture period. Based on this, the 6-day cells were selected for further experiments. Figure 4f, g showed that endogenous USP11 expression was successfully knocked down. The effect of downregulation of USP11 on mHtt expression in the cells was examined. A large amount of mHtt aggregates appeared in the Q74 cells (Fig. 5a, b). Downregulation of USP11 reduced the expression of mHtt aggregates. Compared to the Q23 cell, the viability of the Q74 cell was decreased, but it was improved by USP11 silencing (Fig. 5c). The results of TUNEL staining and flow cytometry showed a large number of TUNEL positive cells and Annexin V-PI staining positive cells in Q74 cells, but they were reduced by USP11 silence (Fig. 5d, e). The expressions of apoptosis-related markers Bax and Bcl-2 were decreased and increased by USP11 silencing in Q74 cells, respectively (Fig. 5f). These evidences suggested that USP11 knockdown inhibited Q74 cell apoptosis.

### Downregulation of USP11 prevented mitochondrial dysfunction in Q74 cells

**Fig. 6:**
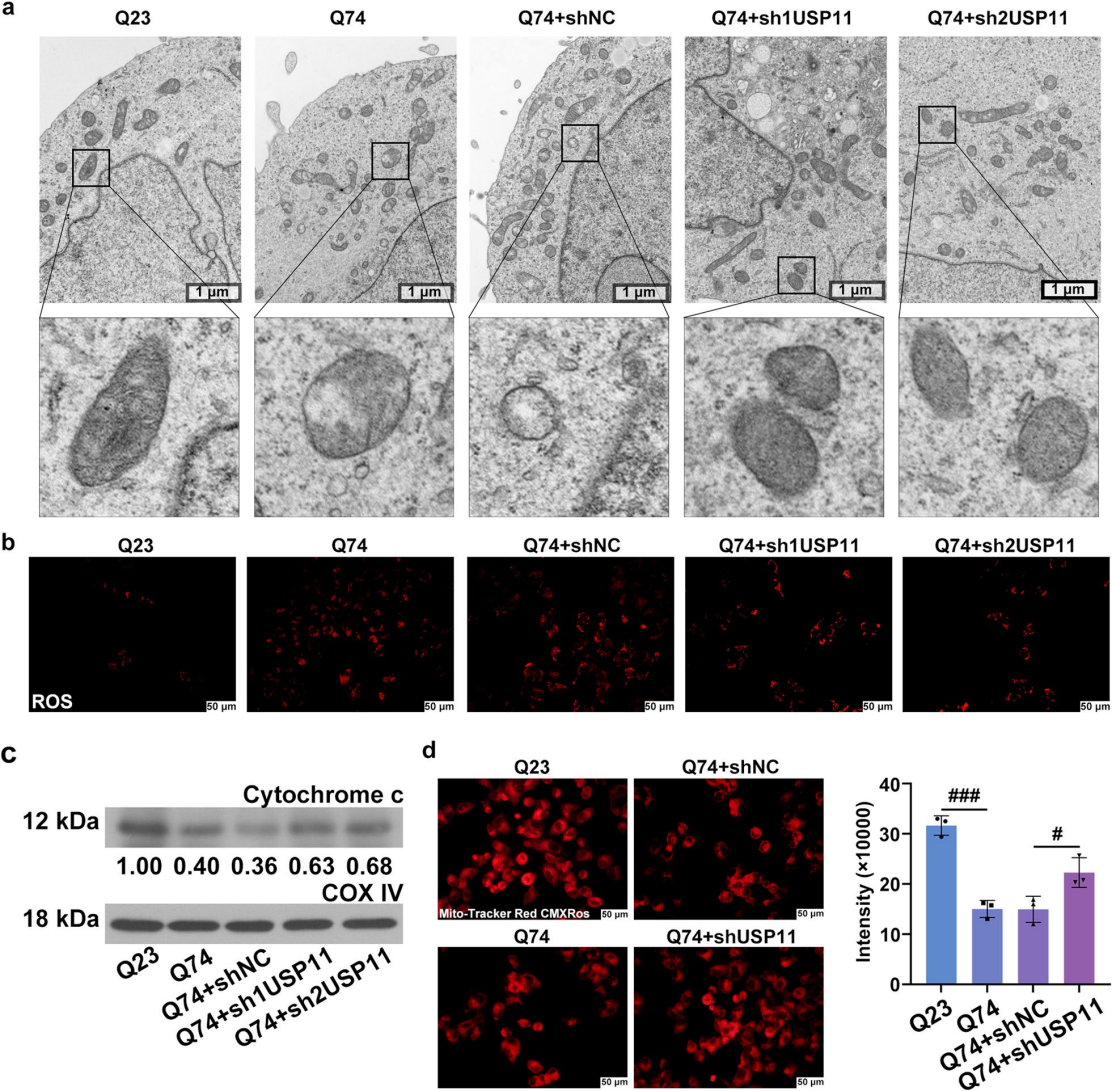
**a** The mitochondria were observed using the transmission electron microscope. **b** The level of ROS was detected using MitSox staining. **c** The cytochrome c protein level in mitochondria was analyzed using western blotting. **d** The mitochondrial membrane potential was analyzed by Mito-Tracker Red CMXRos staining. ^#^p < 0.05, ^###^p < 0.001. ROS: Reactive Oxygen Species

The damaged mitochondria, especially mitochondrial cristae deletion, can be observed in the Q74 cells (Fig. 6a). Silencing of USP11 protected the mitochondrial structure. The level of ROS in the mitochondria was detected using MitoSox staining (Fig. 6b). The production of ROS in the Q74 cells was increased, but it was decreased by USP11 silencing. The decreased expression of cytochrome c in the mitochondria of Q74 cells was promoted by USP11 downregulation (Fig. 6c). In addition, the mitochondrial protection effect of USP11 knockdown also showed that the mitochondrial membrane potential was increased (Fig. 6d).

### Knocking down USP11 affected the protein expression in Q74 cells

**Fig. 7:**
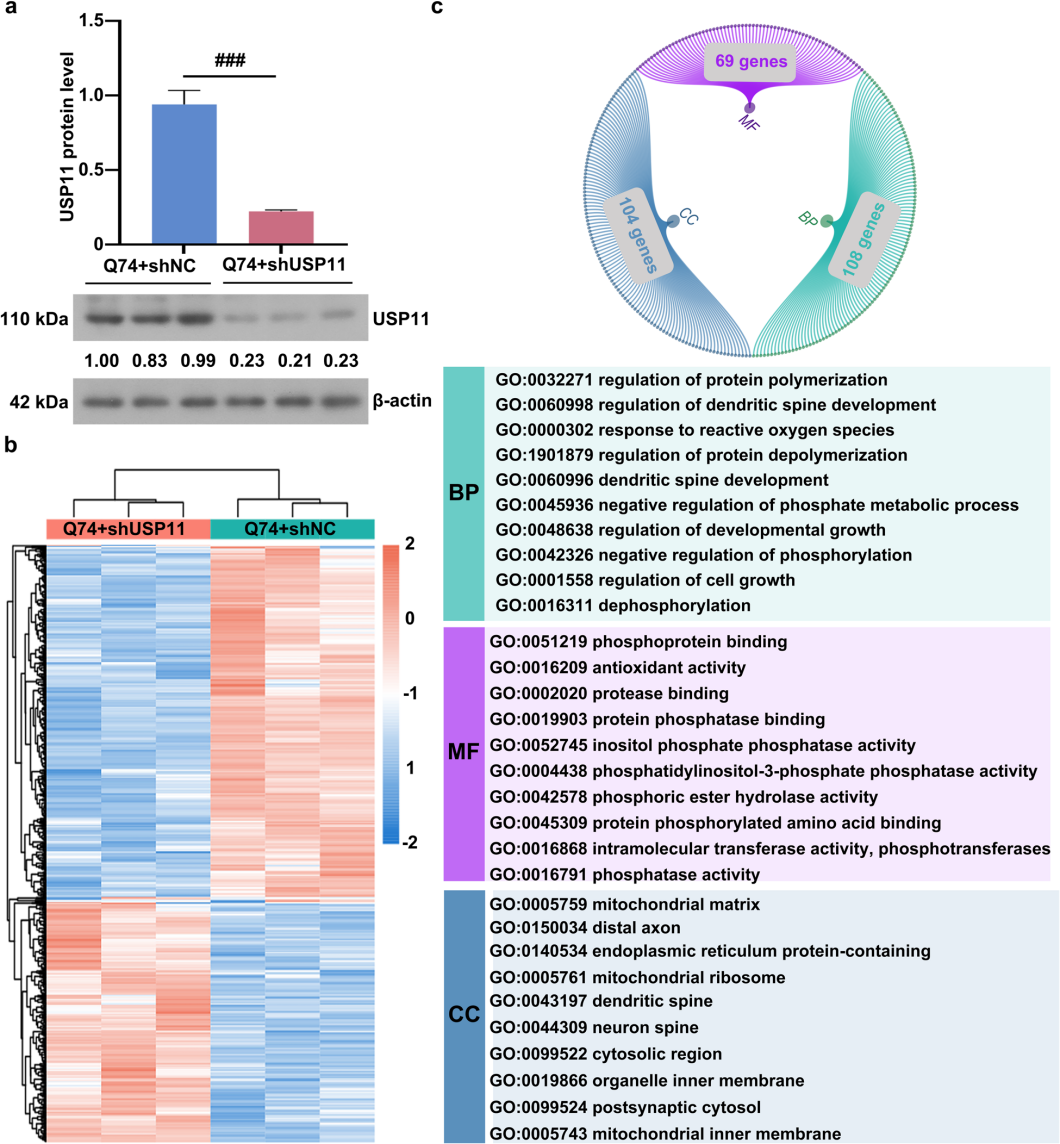
**a** The USP11 protein level was analyzed using western blotting. **b** The heatmap of differential proteins. **c** The differential proteins were analyzed using GO annotation and enrichment. USP11: Ubiquitin specific peptidase 11; GO: gene ontology

**Fig. 8:**
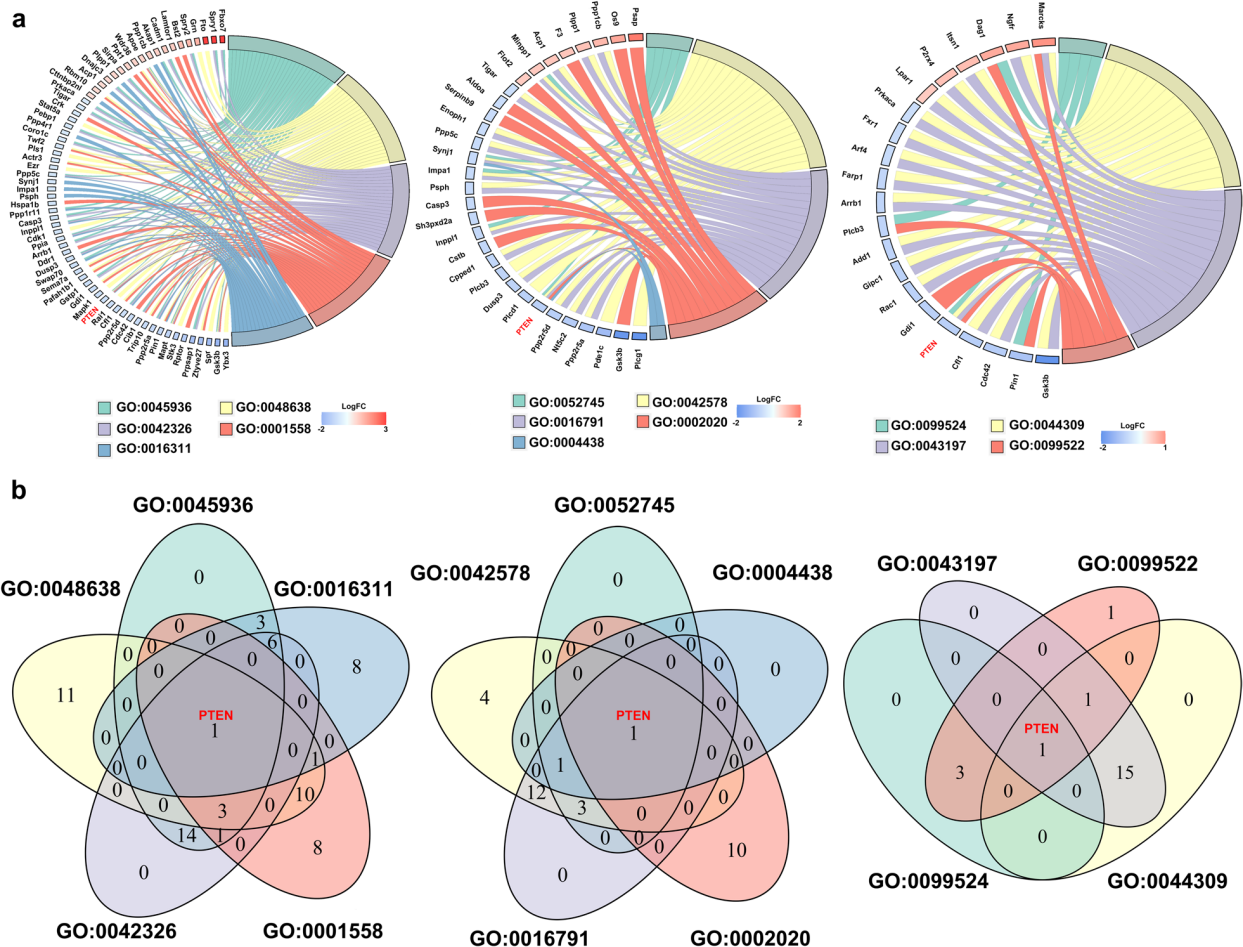
**a** The proteins involved in phosphorylation and cell growth in the HD cell model were analyzed using GO annotation and enrichment. **b** The common proteins involved in both phosphorylation and cell growth were presented via the Venn diagram. HD: Huntington’s Disease; GO: gene ontology

Figure 7a showed a decrease in the level of USP11 in the Q74-shUSP11 cell. The proteomic analysis results were shown in Fig. 7b. Silencing USP11 altered protein expression in Q74 cells. Compared with Q74-shNC cells, 669 differentially expressed proteins (FC > 1.5/FC < 1/1.5 & p < 0.05) were identified in Q74-shUSP11 cells, including 273 up-regulated proteins and 396 down-regulated proteins. GO enrichment analysis was performed for these differentially expressed proteins. GO enrichment results (Fig. 7c) indicated that the knockdown of USP11 altered the biological processes related to the regulation of protein aggregation, neuronal development, and phosphorylation. The molecular functions that regulate protein phosphorylation are primarily changed. Various differential proteins were localized in neurons, mitochondria, or the endoplasmic reticulum. The differentially expressed proteins primarily involved include the up-regulated proteins Lpar1, Hspd1, Sirpa, Itsn1, Apoe, Ngfr, Marcks, and Psap, as well as the down-regulated proteins Gsk3b, Rdx, Mapt, Pin1, Ppp2r5a, Cib1, Cdc42, Ppp2r5d, Cfl1, PTEN, Mapk1, Gstp1, Pafah1b1, Rac1, Dusp3, Add1, Arrb1, Inppl1, Casp3, Ppp5c, Actr3, Twf2, Crk, Tigar, and Prkaca. Based on the above enrichment results, the proteins involved in both phosphorylation and cell growth in the HD cell model was screened to further explore the role of the phosphorylation-related target gene regulated by USP11 in HD (Fig. 8a). PTEN, which was common to all GO terms (GO:0045936, GO:0048638, GO:0042326, GO:0001558, GO:0016311, GO:0052745, GO:0042578, GO:0016791, GO:0002020, GO:0004438, GO:0099524, GO:0044309, GO:0043197, GO:0099522), was found (Fig. 8b).

### Knockdown of USP11 promoted the degradation of PTEN

**Fig. 9:**
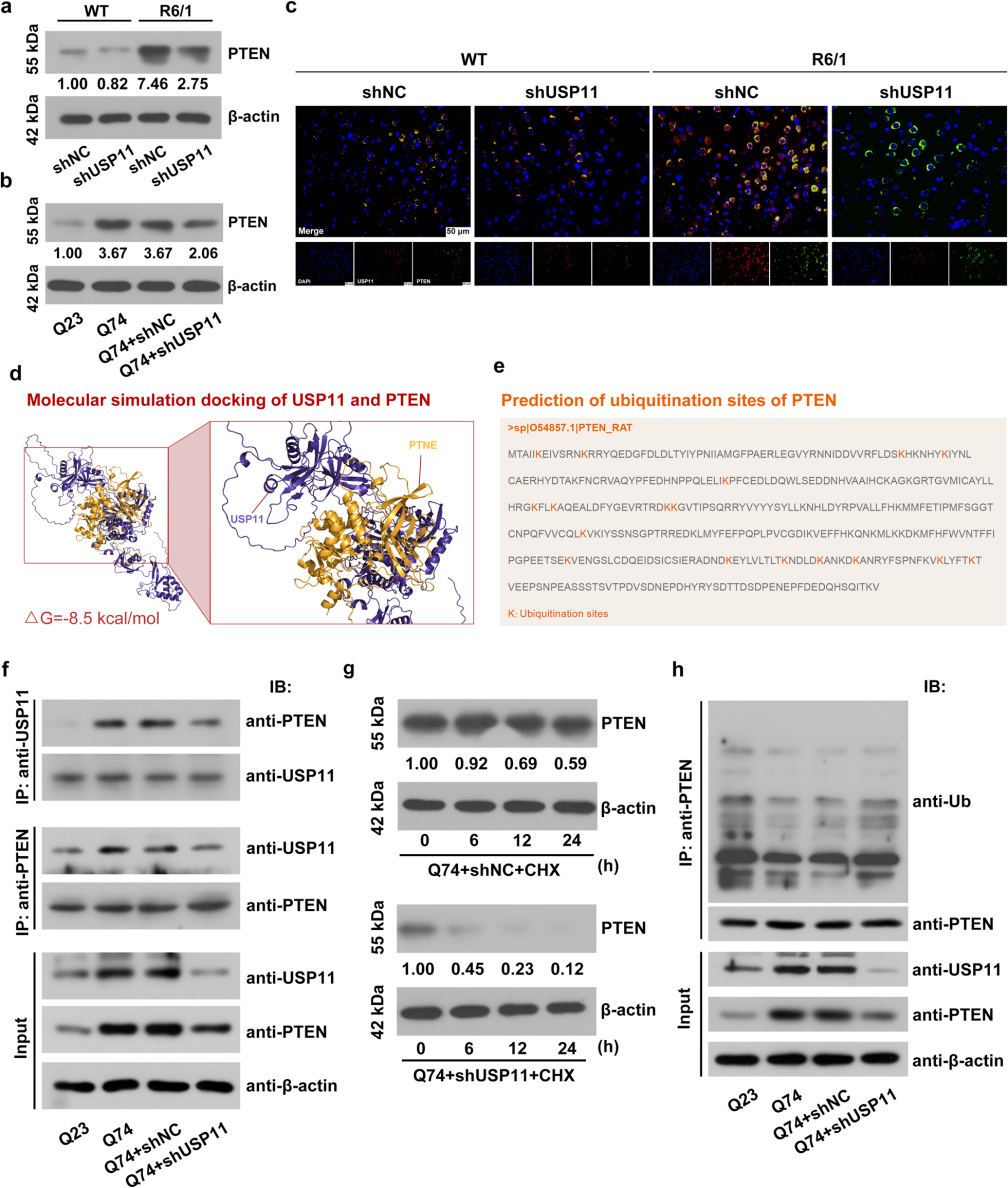
**a**, **b** The PTEN protein level was analyzed using western blotting. **c** The location of USP11 and PTEN in the striatum tissue was detected using immunofluorescence staining. **d** Molecular simulation docking of USP11 and PTEN. **e** Prediction of ubiquitination sites of PTEN. **f** Interaction of USP11 and PTEN was analyzed using co-immunoprecipitation. **g** The PTEN protein level was analyzed using western blotting. **h** Ubiquitination levels of PTEN was analyzed using co-immunoprecipitation. USP11: Ubiquitin Specific Peptidase 11; PTEN: Phosphatase and Tensin Homolog; CHX: cycloheximide

The expression of PTEN was increased in R6/1 mice, and USP11 knockdown positively regulates the expression of PTEN (Fig. 9a). Similar results were observed in cells, and USP11 knockdown reduced PTEN expression in Q74 cells (Fig. 9b). In the striatum tissues, the colocalization of USP11 and PTEN was observed (Fig. 9c). The relationship between USP11 and PTEN in the Q74 cells was then investigated. The results of molecular simulation docking showed that USP11 spontaneously bound to PTEN (Fig. 9d). The existence of multiple potential ubiquitination modification sites on the amino acid sequence of PTEN was predicted by MusiteDeep software (Fig. 9e). The Co-IP results revealed that USP11 and PTEN interact with each other at the protein level (Fig. 9f). In order to verify the hypothesis that USP11 may be involved in the protein stability of PTEN, the production of proteins in the Q74 cells was restrained by treatment with CHX (Fig. 9g). The degradation of PTEN protein was promoted by USP11 silencing. The detection of ubiquitination modification of PTEN revealed that USP11 silencing increased the ubiquitination modification level of PTEN (Fig. 9h), indicating that USP11 stabilized PTEN expression by deubiquitination modification.

### Knockdown of USP11 prevented mitochondrial dysfunction and apoptosis by inhibiting PTEN expression

**Fig. 10:**
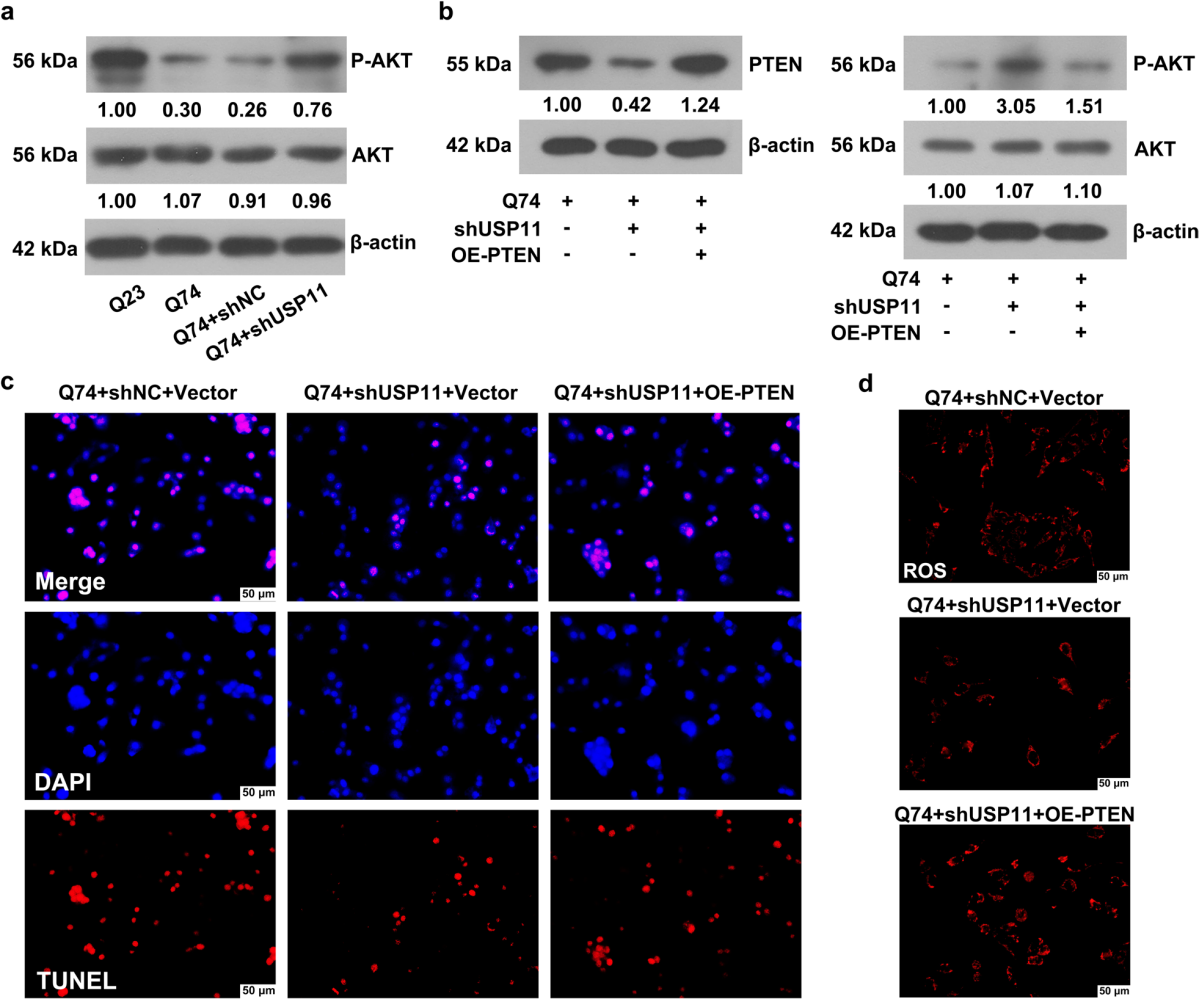
**a** The protein level of p-AKT and AKT was analyzed using western blotting. **b** The protein level of PTEN, p-AKT, and AKT was analyzed using western blotting. **c** The cell apoptosis was analyzed using a TUNEL assay. **d** The level of ROS was detected using MitSox staining. PTEN: Phosphatase and Tensin Homolog; AKT: AKT Serine/Threonine Kinase; p-AKT: Phospho-AKT Serine/Threonine Kinase

The triggering of the AKT signaling pathway by USP11 was examined. The expression of p-AKT was reduced in the Q74 cells, but the downregulation of USP11 increased the phosphorylation of AKT (Fig. 10a). This suggested that knocking down USP11 inhibited PTEN expression, and activated its downstream AKT signaling pathway. Next, rescue experiments were conducted to explore the role of PTEN in the regulation of USP11 in Q74 cells. USP11 knockdown activated the AKT signaling pathway, and inhibited apoptosis and ROS production (Fig. 10b and d). However, these effects were abolished by overexpression of PTEN.

## Discussion

HD is a devastating disease, and existing treatments mainly center on delivering symptomatic drugs. However, there are currently no available treatments that can improve or prevent HD. As more information was revealed about the pathogenesis of HD, targeted RNA therapy may offer hope for patients with HD. In this study, R6/1 mice from F1 progeny of a cross between male R6/1 mice and female C57BL/6J mice was obtained, and it was observed that the expression of USP11 was elevated in R6/1 mice. USP11 may play a crucial role in HD. The additional research was then conducted to explore the role of USP11 and its downstream protein PTEN in regulating mitochondrial function and neuronal growth during HD in vivo and in vitro. The mechanism may involve the PTEN-AKT signaling pathway. These findings revealed the intricate interactions among USP11, PTEN, mitochondrial dysfunction, and neuronal death in the context of HD. This study provided new insights into targeted RNA therapy for HD.

The present study detected the presence of mHtt aggregates and soluble mHtt in striatal tissues in the brains of 16-week-old R6/1 mice. The increase of mHtt aggregates, a typical feature of HD, was reported in R6/1 mice of the same week age (Ochaba et al. 2019), and this study detected soluble mHtt in R6/1 mice, which is also a marker of HD (Luis-Ravelo et al. 2018). In addition, mHtt aggregates have been reported to be detected in the striatum of R6/1 mice at 10 and 12 weeks of age (Luis-Ravelo et al. 2018; Snyder-Keller et al. 2010), suggesting that R6/1 mice may develop disease before 10 weeks of age, and it is appropriate to use 16-week-old R6/1 mice as HD models in this study. HD is characterized by progressive cognitive impairment and abnormal motor symptoms. Clinical manifestations include slow movement, impaired fine motor skills, cognitive decline, and behavioral or mental changes (Rüb et al. 2016). In this study, the motor ability and working memory of HD mice were assessed using the open field test and Y-maze. R6/1 mice (16-week-old) had significantly weaker locomotor ability and engaged in fewer walking behaviors compared to WT mice. Although open-field results in 16-week-old R6/1 mice have been rarely reported, both 10-week-old and 12-week-old R6/2 mice have been reported to walk shorter distances in the open field (Laprairie et al. 2019; Menalled et al. 2010). This also indirectly supports the finding that 16-week-old R6/1 mice reported in this study showed significant motor disturbance. In addition, it was found that distances in the central region of the arena R6/1 mice walked was shorted, indicating that R6/1 mice exhibited anxiety-like behavior (Zeef et al. 2012). The 16-week-old R6/1 mice were observed to lack spontaneous alternations and to have a reduced preference for new arms, which was consistent with previous findings (Pietropaolo et al. 2015). In histopathology, the enlargement of the lateral ventricles and severe atrophy of the striatum are neuropathological features of HD (Paulsen et al. 2006). In this study, H&E staining of the striatum of HD mice revealed a severely atrophied striatum and lateral ventricular enlargement, consistent with previous reports (Ramírez-Jarquín et al. 2022). To explore the role of USP11 in HD, USP11 expression in the striatum was knocked down by intracranial injection of AAV carrying a USP11-targeted recombinant plasmid. USP11 silencing was found to improve walking behavior, anxiety-like behavior, and working memory in R6/1 mice. Pathological examination found that USP11 silencing alleviated striatal atrophy and reduced mHtt aggregates in the striatum. These evidences suggest that USP11 silencing may antagonize HD development.

Based on the above phenomena, the molecular mechanism of USP11 in HD was further explored. In this study, TUNEL-positive cells were detected in the striatum of R6/1 mice aged 16 weeks. A pathological study showed that a large number of TUNEL-positive cells were detected in the striatal tissue of patients with lower-grade HD, while this was reduced in higher-grade cases (Thomas et al. 1995). In this study, the lower-grade HD might be simulated and therefore detected TUNEL-positive cells. Earlier reports showed no apoptotic morphological features in the striatum of HD mice (Davies et al. 1999). This means that the TUNEL-positive cells reported in this study may not represent neuronal apoptosis. TUNEL is an effective means of detecting DNA fragmentation, however, TUNEL positivity is not definitive evidence of apoptosis. Although there is currently no evidence that HD causes actual apoptosis of neuronal cells in the striatum, based on this study and previous reports, there is no denying the presence of TUNEL-positive cells in the striatum. TUNEL remains a valuable assay for HD cell death. However, TUNEL may not be used to mark apoptosis alone and need to be combined with morphological changes. Elevated activated Caspase-3 expression has been reported in the striatal tissues of HD mice with striatum atrophy (Gardian et al. 2005). The presence of TUNEL-positive cells and increased expression of related markers indicate that neuronal cells in the striatum might be subjected to stress, which caused the striatum atrophy, but not be heading for apoptosis.

However, different conclusions may be drawn in PC12 cells. The Q74 cells used in this study have been reported to decrease significantly in cell number 48 h after induction (Karachitos et al. 2016). Elevated cleaved-caspase 3 and TUNEL-positive cells were reported in Q74 cells (Sundaram et al. 2019). Furthermore, in Q74 cells, the accumulation of mHtt leaded to apoptosis, which was demonstrated by Annexin V-PE/7-AAD staining combined with flow cytometry (Dong and Cong 2021), which was consistent with the results of this study. Annexin V-PE/7-AAD staining positive indicated cell membrane exposure and increased cell membrane permeability, which is a morphological feature of apoptosis. Combined with the change of apoptosis marker protein Bcl-2 family expression, apoptosis can be explained. In addition, actual cell loss and increased cleaved Caspase 3 expression were detected in striatal neurons differentiated from HD patient-derived IPSC (Virginia et al. 2012). On the other hand, studies have shown that HIP-1 induces the apoptotic morphology of human neuronal precursor cells NT2 and increases the activity of caspase 3 (Hackam et al. 2000). The proteomic analysis in this study showed that USP11 knockdown reduced the expression of HIP-1, which may be a potential pathway for USP11 knockdown to inhibit Q74 cell apoptosis. USP11 promotion of apoptosis has been reported in rat microglia after intracerebral hemorrhage, involving the deubiquitination of substrate protein Beclin 1 by USP11, which is consistent with the role of USP11 in neurological diseases reported in this study USP11 (Zhang et al. 2021).

There are few reports on the difference between R6/1 mouse model and PC12 cells induced to express CAG repeat amplification sequence. Based on the adverse effect of mHtt aggregates on neurons, we hypothesize that R6/1 mice and PC12 cells provide different protein expression environments for mHtt, which leads to the possibility that mHtt induces cell death through different mechanisms in vivo and in vitro. In addition, environmental differences between in vivo and in vitro conditions lead to distinct external stimuli being received by striatal neurons and PC12 cells. The complex environment within the body can create conditions that damage striatal neurons while simultaneously activating the body’s self-protection mechanisms to offer compensatory support. These combined factors may result in striatal atrophy rather than actual apoptosis. In vitro, neurons are solely exposed to the toxic effects of mHtt, which represents a simpler stimulus compared to in vivo conditions and may lead to different outcomes than those observed in vivo.

Neurons are high-energy-demanding cells, and their normal function is closely related to mitochondrial function. Mitochondrial damage was observed both in vivo and in vitro in the HD model, which was reflected in increased ROS levels and decreased mitochondrial membrane potential. Previous studies have shown that ROS accumulation as well as redox imbalances are observed in the brains of HD mice (Sadagurski et al. 2011). ROS is an important factor that causes neuronal cells to be stressed and even die (Zheng et al. 2018), which explains the TUNEL-positive neurons and the apoptosis of Q74 cells detected in this study. This study found that USP11 knockdown alleviated oxidative stress and protected mitochondrial function. USP11 has been reported to enhance oxidative stress in neuronal cells, thereby leading to ferroptosis (Rong et al. 2022). In endothelial cells receiving radiotherapy, USP11 promoted ROS production (Tang et al. 2024). These reports suggested the role of USP11 as a facilitator of oxidative stress.

Label-free proteomic analysis revealed that silencing USP11 significantly altered protein expression in Q74 cells. The levels of proteins associated with neuron growth, protein polymerization, and protein phosphorylation were significantly altered. Protein phosphorylation is closely related to HD pathogenesis (White et al. 2022). It has been reported that there was a widespread disorder of protein phosphorylation in the cerebral cortex in mice before the onset of HD (Mees et al. 2022). PTEN, which was involved in both the phosphorylation pathway and cell growth, was highly expressed in the striatum of HD mice and Q74 cells, and was downregulated by USP11 silencing. In this study, it was revealed that USP11 knockdown promoted the ubiquitination degradation of PTEN, and activated the AKT signaling pathway. In hippocampal cells, blocking of the PTEN/AKT signaling pathway inhibited ROS production and apoptosis (Zhu et al. 2006). In this study, overexpression of PTEN prevented AKT signaling activation in Q74 cells, thereby reversing the inhibition of apoptosis and ROS by USP11 knockdown, which was consistent with previous findings and suggested that the USP11/PTEN/AKT axis was involved in the regulation of oxidative stress and neuronal damage during HD progression. In addition, overexpression of PTEN has been shown to induce glioblastoma death by inhibiting the activation of AKT signaling pathway, leading to mitochondrial dysfunction (Bao et al. 2019). The activation of the PTEN/AKT signaling pathway, triggered by USP11, may lead to mitochondrial dysfunction and oxidative stress, creating a detrimental environment for neuronal cells and consequently promoting neuronal cell damage. In tumor cells, USP11 has been reported to inhibit tumor progression through a PTEN-dependent mechanism involving apoptosis (Park et al. 2019). However, in HD, the function of the USP11/PTEN/AKT axis was not revealed, which is an innovative point in this study. In addition, in previous reports on HD, several genes have been shown to have potential as therapeutic targets for HD, including BDNF and Sirt1 (Xie et al. 2010; Jiang et al. 2011). This study focused on USP11, a deubiquitinating enzyme, which was a valuable point of this study. Deubiquitinating enzymes act on many substrate proteins, and USP11 has been reported to regulate the stability of PML, P53, E-cadherin and other substrate proteins through deubiquitination (Wu et al. 2014; Ke et al. 2014; Qian et al. 2024). Among them, P53 has been reported to promote mitochondrial dysfunction and neurobehavioral abnormalities in HD (Bae et al. 2005). It can be speculated that these known or unknown proteins may be important targets in addition to PTEN that mediate the progression of USP11 regulation of HD. This provides a research basis and broad vision for further exploring the molecular mechanism mediating HD pathogenesis.

Last but not least, at present, many kinds of HD mouse models have been reported, including KI140CAG, zQ175 and other knock-in mice in addition to the widely used transgenic models such as R6 strain (Pépin et al. 2016; Smith et al. 2023). The pathogenesis of this HD model is the expansion of CAG repeat sequence, but the copy number of CAG is different. As long as the animal model expressed mHtt through CAG sequence expansion, it has the potential to be used to explore HD progression in vivo. Therefore, we did not utilize multiple animal models, such as knock-in mice. Notably, the knock-in mice showed a characteristic of slow progression of symptoms, which may be more similar to human HD pathology (Menalled and Chesselet 2002). This is a limitation of this study. R6/1 mouse was a widely used mouse model in the study of Huntington’s disease (Espina et al. 2023; Rodríguez-Urgellés et al. 2023), and this model mimics a key pathogenic factor of HD, CAG sequence expansion and mHtt accumulation. Therefore, in this study, the role of the USP11/PTAN/AKT axis revealed in R6/1 mice has important implications for HD prevention and treatment. For cell model, iPSC-derived striatal cultures from patients with HD could be a cell model directly relevant to human patients. Similar to animal models, in this study, PC12 cells, which were induced to express a fragment of HTT exon 1 containing 74 CAG repeats (Q74), also exhibits a key feature of Huntington’s disease, CAG sequence amplification. This cell model has been widely used in HD-related studies (Voelkl et al. 2023; Sun et al. 2024). In addition, this cell model has the advantage of having a suitable negative control cell Q23. Q23 and Q74 cells differed only in the number of CAG copies, which mimics the fundamental differences between healthy individuals and HD patients and helps to unify variables in this study. On the basis of this study, further exploration of the role of USP11 in iPSC-derived striatal cultures from patients with HD may provide more sufficient evidence for preclinical studies.

## Conclusion

The present paper provides important evidence that USP11, when knocked down, promotes PTEN degradation and activates AKT signaling pathway, thereby alleviating mitochondrial dysfunction and relieving oxidative stress. This relieves the pressure on neuronal cells, which reduces neuronal damage. This study demonstrates that the USP11-PTEN-AKT axis may represent a promising new therapeutic target for HD.

## Data Availability

No datasets were generated or analysed during the current study.
